# A Rare Case of Vascular Proliferation in the Mandible of a Juvenile Horse

**DOI:** 10.3389/fvets.2020.573540

**Published:** 2020-11-11

**Authors:** Eva Leitzen, Sebastian Stumpf, Claudia Zimmermann, Astrid Bienert-Zeit, Maren Hellige, Wolfgang Baumgärtner, Christina Puff

**Affiliations:** ^1^Department of Pathology, University of Veterinary Medicine Hannover, Hannover, Germany; ^2^Equine Clinic Großmoor, Adelheidsdorf, Germany; ^3^Clinic for Horses, University of Veterinary Medicine Hannover, Hannover, Germany

**Keywords:** angiomatosis, congenital, equine, horse, mandibular mass, vascular proliferation

## Abstract

A fast growing, circumscribed, unilateral swelling of the right mandible of a juvenile horse was observed. Within few weeks, the continuously growing mass reached dimensions ranging from 7 to 10 cm in diameter and resulted in loss of the first deciduous premolar of the affected side. The animal was euthanized due to lesion progression. Histologically the mandibular swelling consisted of numerous variably sized vascular structures, partly filled with erythrocytes and embedded in a loosely arranged fibrous stroma within the medullary cavity of the mandible. Juvenile mandibular angiomatosis was diagnosed. To the authors' knowledge this is the first description of this rare entity in the mandible of a foal.

## Introduction

Swellings and masses of the equine mandible predominantly arise from dental diseases such as apical infections of cheek teeth, but can also be caused by trauma as well as non-neoplastic or neoplastic proliferations (1–3). Most mandibular neoplasms, already rare in itself, originate from osteogenic or odontogenic tissues whereas other tumors are only sporadically reported in literature (1, 4–9). Moreover, a clear distinction between neoplasms and tumor like-lesions, for example granulation tissue formation, epulides, and vascular proliferations can pose a diagnostic challenge. Vascular tumors can be differentiated according to their cellular origin (lymphatic endothelium, blood vessel endothelium, perivascular wall tumors in small animals) as well as their dignity (e.g., hemangioma/hemangiosarcoma).

Tumor like vascular proliferations (e.g., vascular hamartomas, angiomatosis/hemangiomatosis, aneurysmal bone cysts) are rarely reported in animals, especially with a view to the mandibular region (10). Furthermore, the used terminology is often complicated due to frequent overlap between simple epithelial proliferations and benign tumors of endothelial origin like hemangiomas or lymphangiomas (10). Accordingly, the term angiomatosis has been used ambivalently in literature to describe both, neoplastic, as well as malformative, proliferative vascular lesions (11–13). Cases of mandibular angiomatosis have been reported in young animals before, including a 2-month-old Holstein calf and a 6-month-old dog from Canada and Spain, respectively (14, 15). Similar lesions have been observed in the orbit, the meninges, ovaries, intestine, and skin of horses but, to the authors' knowledge, have never been observed within the mandible (11–13, 16). Therefore, this report describes the first case of a juvenile, mandibular angiomatosis in a horse.

## Case Presentation and History

A 178 kg, about 4 months old, Hanoverian colt was presented to a local horse clinic with a fast-growing mass of the right mandible with consecutive loss of the first deciduous premolar (Pd2). A 3 x 3 x 1.5 cm sized, indolent swelling had already been present at birth. During the following 6–8 weeks until euthanasia, the animal was clinically and radiologically examined twice. A rapid growth with consecutive loss of Pd2 was noted. The animal remained in good general condition for the entire investigation period. Feed intake, however, became increasingly impaired over time due to the progressive growth of the oral mass. Since the animal did not reveal any increased sensitivity or painfulness, no analgetic treatment or other medication was performed. A biopsy sample from the right mandible was taken by the local veterinarian and sent to the Department of Pathology, University of Veterinary Medicine Hannover for histological examination. Afterwards, the animal was transferred to the clinic for horses, University of Veterinary Medicine Hannover for further imaging techniques.

## Diagnostic Assessment

For computed tomography (CT) examination the foal was positioned in left lateral recumbency on a stationary examination table. CT was performed using a Brilliance™CT—Big Bore Oncology 16 slice Scanner (Philips Medical Systems, Best, the Netherlands). The entire head of the colt was scanned with an axial scan-mode with 140 kV, 500 mAs, slice thickness of 1.5 mm. Scanning commenced in a soft tissue window (WL: 58 and WW: 400) followed by reconstruction in a bone window (WL: 300, WW: 2800).

CT revealed a thickening of the cortex starting at the level of the third incisor (Id3) of the right mandible with an interruption of the bony contour (4 x 9 mm) to the buccal side 2 cm caudal of Id3. Over a length of 14 cm, the right mandible was thickened up to 5 cm with partial cortical thickening, cortical disruption at the buccal and lingual side and solid periosteal new bone formation. Pd2 was missing and an expansile mass (15 x 4 x 7 cm) with loss of normal trabecular structure, causing a disruption of the dorsal border of the mandible, extending into the right side of the oral cavity, overtopping the level of the mandible and the mandibular premolar teeth, and displacing the tongue to the left was present. The mass contained irregular mineralized opacities that were more prominent rostrally and showed heterogeneous multilobulated soft tissue appearance with a mean HU of 37. The right mandibular lymph node was markedly enlarged.

Due to the progressive nature of the lesion and the increasing physical impairment of the horse, the animal was euthanized and submitted for full post-mortem examination.

During necropsy a representative spectrum of tissue samples of various organs including mandibular bone and mass, overlying skin as well as associated lymph nodes (*Lnn. mandibulares*, right *Ln. retropharyngealis*) and tongue was taken and fixed in 10% neutrally buffered formalin for histochemical and immunohistochemical investigations. Formalin fixed tissue was routinely embedded in paraffin wax (formalin and paraffin embedded material; FFPE) and sectioned on a microtome at 2 μm thickness and stained with hematoxylin and eosin (HE). Osseous tissue was decalcified using 5% nitric acid (HNO_3_). Moreover, tissue sections from the oral mass were stained using Heidenhain's azan trichrome stain for visualization of collagen fibers and alcian blue stain (0.1%; pH 2.5) for detection of mucinous components (17, 18). Immunohistochemistry (IHC) was used for the detection of vimentin (mesenchymal cells; clone V9, 1:100, Dako, Hamburg, Germany; catalog no. M0725), alpha-smooth muscle actin (smooth muscle cells; alpha-SMA; 1:200 Dako, Hamburg, Germany; catalog no. M0851), CD31 (endothelial cells; 1:100; Acris, Herford, Germany; catalog no. AP15436PUM), factor VIII-related antigen (endothelial cells; FVIIIrA; 1:500; Dako, Hamburg, Germany; catalog no. A0082) as well as Prospero homebox protein-1 (lymphatic endothelial cells; Prox-1; 1:200; ReliaTech, Braunschweig, Germany; catalog no. 102-PA30S) and carried out as described before (19).

**Figure 1:**
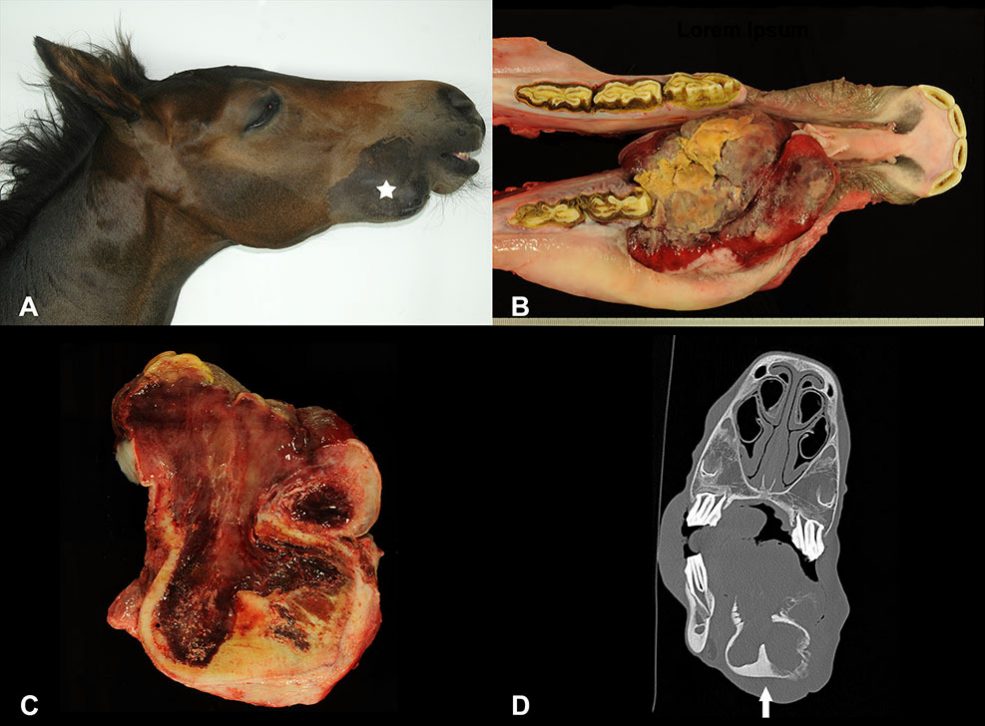
Swelling on the right mandible (**A**; white star) of a foal, showing extensive growth and consecutive loss of the first deciduous premolar **(B)**. The cut surface was reddish and of gelatinous consistency **(C)**. Transverse CT image of the oral mass involving the right mandible (**D**; arrow).

At necropsy the animal was in a good nutritional condition. An ~10 x 7 x 7 cm large, firm, elastic swelling of the right mandible (Figure 1A, white star) localized at the diastema was found. The mass was covered by gingiva and caused a protrusion of the mandibular bone with consecutive loss of Pd2 (Figure 1B). The multinodular oral growth exhibited extensive ulceration. Moreover, it compressed and displaced the surrounding osseous tissue. The cut surface of the proliferation was homogenously red and of gelatinous consistency (Figure 1C). Corresponding changes were also detected in CT images (Figure 1D). On the right side of the tongue, affecting the middle to caudal third, there was a focal, brownish, moderately hyperkeratotic area of ~10 x 2 cm. At the left cranial aspects of the tongue, there were multifocal ulcerations, most likely resulting from trauma due to tooth bites. The right mandibular lymph node measured 8 x 2.5 x 2.5 cm. Moreover, there was a 6 x 1 x 0.4 cm sized ulceration at the margo plicatus of the stomach.

**Figure 2:**
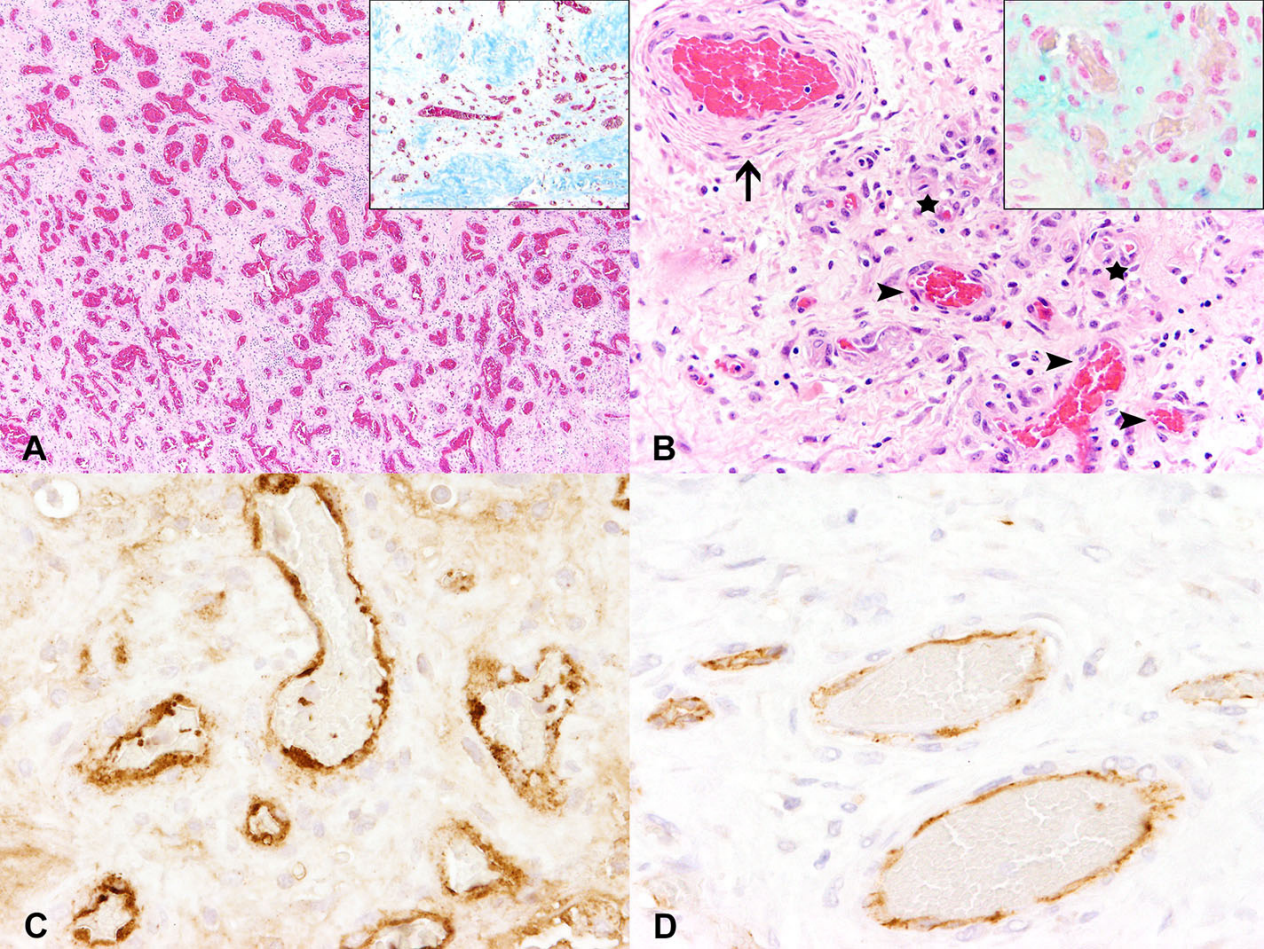
Histochemically and immunohistochemically, the swelling was composed of a dense meshwork of vascular structures of variable calibers partly divided by collagenous fibers (**A**; inset shows a representative section in Heidenhains's trichrome stain). Predominantly thin-walled, venous vessels (arrowheads) and fewer small capillary structures (stars) with single interspersed thick-walled, arterial vessels (arrow) could be detected within a slightly mucinous stroma (**B**; inset shows a representative section in alcian blue stain). Vascular walls stained highly positive for factor VIII-related antigen **(C)** as well as for CD31 **(D)**, indicating a proliferation of blood vessels.

Histologically, a focally extensively ulcerated vascular proliferation consisting of variably sized vessels embedded in a loosely arranged, edematous, slightly mucinous, and collagenous stroma (Figures 2A,B) was observed. Alcian blue and Heidenhains's trichrome staining served to confirm the mucinous and collagenous nature of the stroma. Predominantly venous, thin-walled vascular structures, characterized by large lumina ranging from ~10 μm up to 200 μm in diameter, were found within the mass. The vast majority was filled with variable numbers of erythrocytes, partly containing fibrin thrombi. Vascular walls were lined by 1–2 layers of concentrically arranged, elongated cells, surrounding well-differentiated, sometimes slightly plump endothelial cells. Multifocally, vascular structures were highly branched, partly forming anastomoses and lateral protrusions, resembling aneurysms. Occasionally (~2 per 10 high power fields, magnification 400x), arterial-like vessels, surrounded by more than 2 concentrical cell layers with a lumen measuring up to 150 μm in diameter were detected (Figure 2B). Endothelial cells did not exhibit noticeable anisocytosis or -karyosis or an increased mitotic rate. The vessels within the mass stained moderately positive for vimentin, and highly positive for FVIIIrA (Figure 2C), CD31 (Figure 2D) and alpha-SMA. Prox-1 was only detected in a very small subset (<1 per 10 high power fields, magnification 400x) of optically empty vascular structures. Multifocally within the proliferation there were areas of newly formed woven bone and an increased osteoclastic activity at the transition zone to the preexisting mandibular bone.

The focal brownish area at the tongue exhibited a moderate parakeratotic hyperkeratosis. The moderate to severe, ulcerative glossitis of the left side and the ulcerative stomatitis were confirmed. Furthermore, a severe follicular hyperplasia was present in the right mandibular lymph node.

Alterations at the tongue were interpreted as direct results of an impaired masticatory movement due to the oral mass. Changes of the mandibular lymph node were interpreted as reactive changes due to the reported inflammatory events within the oral cavity. The gastric ulcer most likely represents a stress-related alteration.

## Discussion

Besides traumatic events and inflammatory processes, mandibular swellings in horses can be caused by non-neoplastic or neoplastic growths (3). One more frequently seen oral tumor-like lesion in young horses is equine juvenile mandibular ossifying fibroma (OF), a fibro-osseous growth that commonly affects horses less than one year of age (20–22). Macroscopically, OF is barely distinguishable from the presented vascular proliferation. Histologically, however, OF shows characteristic features with proliferation of a cell-rich fibroblastic stroma next to osteoblastic cells surrounding islands of osteoid and bone. Therefore, histologic examination served as major distinguishing criterion to exclude this diagnosis in the presented case. Squamous cell carcinoma is probably the most common neoplasm at this site but is usually characterized by invasive growth of epithelial tumor cells (6). Tumors of the skeletal system are rare in horses, more frequently found in the axial than the appendicular skeleton and—for the most part—preferentially occur in adult and older animals (22). However, osteoma, osteosarcoma, osteoblastoma, chondrosarcoma, and fibrosarcoma are reported to occur in the mandible (6). Odontogenic tumors, rare neoplasms in animals by itself, but more frequently seen in horses, can occur as ameloblastoma, cementoma, and mixed-cell tumors (6, 23). Ameloblastomas represent the most common odontogenic tumors in horses with reports of affected horses ranging from neonatal age to >15 years of age, characterized by a proliferation of palisading and stellate-shaped epithelial tumor cells (22). Other possible tumors involving the oral cavity include melanoma, oral papilloma, epulis, salivary adenocarcinoma and myxoma/myxosarcoma (6). Non-neoplastic bone masses include, inter alia, fibrous dysplasia, intraosseous epidermoid cysts, aneurysmal bone cysts and actinomycosis (22). In the present case, the mandibular growth was diagnosed as a benign proliferative lesion mainly consisting of thin-walled, erythrocyte filled blood vessels surrounded by a thin layer of alpha-SMA positive smooth muscle cells. Due to the high expression of FVIIIrA and CD31 as well as only occasionally detectable expression of Prox-1, a proliferation originating from lymphatic vessels was excluded. Aside from the before mentioned neoplasms, vascular tumors also constitute rare events in horses, being predominantly located within the skin (24). Lymphangiomas and lymphangiosarcomas are very rare in animals but especially occur as congenital lesions or at a very young age (25). Intraosseous hemangiomas also are classified as extremely rare events, whereas hemangiosarcomas with involvement of osseous structures are described occasionally (26, 27). There is an ongoing debate whether vascular lesions have to be called non-neoplastic or rated as benign tumors resulting in a huge variation regarding terminology between publications (16, 24). An intraosseous hemangioma has been described within the distal phalanx of a yearling thoroughbred colt (28). Differentiation between hemangiomas and vascular hamartomas is frequently based on the proliferation rate and the level of maturity of cells (28, 29). Lacking a harmonized classification system, different terms including hemangiomas as well as lymphangiomas, hamartomas, (hem)angiomatosis, proliferative angioma, arteriovenous malformation, vascular naevus or telangiectatic granuloma have been used (12, 16, 24). The choice of terminology is primarily restricted by the histogenesis of the proliferation. Furthermore, it depends on the histologic appearance, especially on whether there is expansile or infiltrative growth. Finally, diagnosis largely depends on whether the investigators want to emphasize a neoplastic or non-neoplastic as well as a congenital or acquired character. This inevitably leads to a large variety of terms used for marginally different, if not similar proliferations. Especially the term angiomatosis comprises several lesions characterized by a proliferation of vascular tissues. Some specific syndromes, like bovine cutaneous angiomatosis, progressive angiomatosis, or scrotal vascular hamartoma are described in literature, partly equally being referred to as hamartomas, angiomatosis or hemangiomas, which is most probably thanks to their considerable macroscopic and histological overlap (25). The exact etiology and pathogenesis of the vascular malformations presented here remains unclear but seems to be congenital in nature. Due to the already existing entity of juvenile bovine angiomatosis in the mandible and the undeniable resemblance of the two lesions, it is proposed to adopt this term also in horses.

## Data Availability Statement

The raw data supporting the conclusions of this article will be made available by the authors, without undue reservation.

## Ethics Statement

Ethical review and approval was not required for the animal study because the presented case derived from an animal which was submitted for routine diagnostic services (Necropsy for determination of the cause of disease). Written informed consent was obtained from the owners for the participation of their animals in this study.

## Author Contributions

AB-Z, CZ, MH, and SS: clinical investigation. CZ and SS: radiographic examination. AB-Z and MH: computed imaging. EL, CP, and WB: pathological and histological examination. EL and CP: writing—original draft preparation. AB-Z, EL, CP, CZ, MH, SS, and WB: writing—review and editing. All authors contributed to the article and approved the submitted version.

## Conflict of Interest

The authors declare that the research was conducted in the absence of any commercial or financial relationships that could be construed as a potential conflict of interest.

## References

[1] Pirie RS Dixon PM Mandibular tumours in the horse: a review of the literature and 7 case reports. Equine Vet Educ. (1993) 5:287–94. 10.1111/j.2042-3292.1993.tb01055.x

[2] Dixon PM Tremaine WH Pickles K Kuhns L Hawe C McCann J. Equine dental disease part 4: a long-term study of 400 cases: apical infections of cheek teeth. Equine Vet J. (2000) 32:182–94. 10.2746/042516400776563581 10836472

[3] Dixon PM Reardon RJM Equine mandibular growths. Equine Vet Educ. (2015) 27:16–21. 10.1111/eve.12260

[4] Gardner DG. Ameloblastomas in the horse: a critical review and report of an additional example. J Oral Pathol Med. (1994) 23:41–4. 10.1111/j.1600-0714.1994.tb00252.x 8138980

[5] Chandra AMS Buergelt CD Ethell MT. Odontogenic myxoma of the mandible in a filly. J Vet Diagn Invest. (1999) 11:274–7. 10.1177/104063879901100311 10353360

[6] Knottenbelt DC Kelly DF Chapter 12 - oral and dental tumors. In: Baker GJ Easley J editors. Equine Dentistry (Second Edition). Oxford: WB Saunders (2005) 127–48. 10.1016/B0-70-202724-3/50015-3

[7] Greet TR Boys Smith SJ Foote AK Steven WN. Mandibular lymphoma in a three-year-old thoroughbred filly. Vet Rec. (2011) 168:80. 10.1136/vr.c6097 21257588

[8] Carmalt JL Linn KA. Large segmental mandibulectomy for treatment of an undifferentiated sarcoma in a horse. Vet Surg. (2013) 42:433–9. 10.1111/j.1532-950X.2013.01086.x 23432182

[9] Crijns CP Vlaminck L Verschooten F Bergen T De Cock HE Huylebroek F Multiple mandibular ossifying fibromas in a yearling Belgian Draught horse filly. Equine Vet Educ. (2015) 27:11–5. 10.1111/eve.12246

[10] Robinson WF Robinson NA Chapter 1 - Cardiovascular System. In: Maxie MG Saunders WB editors. Jubb, Kennedy & Palmer's Pathology of Domestic Animals: Volume 3. 6th ed St. Louis, MO: Elsevier (2016). p. 1-101.e101 10.1016/B978-0-7020-5319-1.00012-8

[11] McEntee M Summers BA De Lahunta A Cummings J. Meningocerebral hemangiomatosis resembling Sturge-Weber disease in a horse. Acta Neuropathol. (1987) 74:405–10. 10.1007/BF00687221 3687394

[12] Lamm CG Njaa BL. Ovarian and intestinal angiomatosis in a horse. Vet Pathol. (2007) 44:386–8. 10.1354/vp.44-3-386 17491083

[13] Ludwig HC Pucket JD Shaw GC Equine orbital angiomatosis. Equine Vet Educ. (2015) 29:426–30. 10.1111/eve.12520

[14] Richard V Drolet R Fortin M. Juvenile bovine angiomatosis in the mandible. Can Vet J. (1995) 36:113–4. 7728728 PMC1686849

[15] Peña ML Muñoz F Alemañ N González A Pereira JL Nieto JM. Hemangiomatosis associated with osteolysis of the mandible in a dog resembling Gorham-Stout disease in humans. Vet Pathol. (2005) 42:489–91. 10.1354/vp.42-4-489 16006608

[16] Platt H. Vascular malformations and angiomatous lesions in horses: a review of 10 cases. Equine Vet J. (1987) 19:500–4. 10.1111/j.2042-3306.1987.tb02658.x 3504759

[17] Scott JE. Alcian blue. Now you see it, now you don't. Eur J Oral Sci. (1996) 104:2–9. 10.1111/j.1600-0722.1996.tb00038.x 8653492

[18] Udeabor SE Adisa AO Orlowska A Chia P Sader RA Ghanaati S Osteocalcin, Azan and Toluidine blue staining in fibrous dysplasia and ossifying fibroma of the jaws. Alexandria J Med. (2018) 54:693–7. 10.1016/j.ajme.2018.01.001

[19] Jungwirth N Junginger J Andrijczuk C Baumgärtner W Wohlsein P. Plexiform vasculopathy in feline cervical lymph nodes. Vet Pathol. (2018) 55:453–6. 10.1177/0300985817747949 29343196

[20] Morse CC Saik JE Richardson DW Fetter AW. Equine juvenile mandibular ossifying fibroma. Vet Pathol. (1988) 25:415–21. 10.1177/030098588802500603 3212886

[21] Witte S Maxillectomy and mandibulectomy in the horse: indications and necessity of post operative adjunct therapy. Equine Vet Educ. (2014) 26:274–9. 10.1111/eve.12024

[22] Knottenbelt DC Patterson-Kane JC Snalune KL 19 - Bone and dental region neoplasms. In: Knottenbelt DC Patterson-Kane JC Snalun KL editors. Clinical Equine Oncology. London: Elsevier (2015). p. 312–31. 10.1016/B978-0-7020-4266-9.00019-2

[23] Morgan RE Fiske-Jackson AR Hellige M Gerhauser I Wohlsein P Biggi M. Equine odontogenic tumors: clinical presentation, CT findings, and outcome in 11 horses. Vet Radiol Ultrasound. (2019) 60:502–12. 10.1111/vru.12793 31359553

[24] Knottenbelt DC Patterson-Kane JC Snalune KL 20 - Vascular neoplasms. In: Knottenbelt DC Patterson-Kane JC Snalun KL editors. Clinical Equine Oncology. Elsevier (2015). p. 332–41. 10.1016/B978-0-7020-4266-9.00020-9

[25] Hendrick MJ Mesenchymal tumors of the skin and soft tissues. In: Meuten DJ editors. Tumors in Domestic Animals, 5th ed. Ames, IA: John Wiley & Sons Inc (2016). p. 142–75. 10.1002/9781119181200.ch5

[26] Dunkel BM Del Piero E Kraus BM Palmer JE Lin P Wilkins PA. Congenital cutaneous, oral, and periarticular hemangiosarcoma in a 9-day-old Rocky Mountain horse. J Vet Intern Med. (2004) 18:252–5. 10.1111/j.1939-1676.2004.tb00171.x 15058781

[27] Craig LE Dittmer KE Thompson KG Chapter 2 - bones and joints. In: Maxie MG editors. Jubb, Kennedy & Palmer's Pathology of Domestic Animals: Volume 1. WB Saunders (2016). p.16-163.e161. 10.1016/B978-0-7020-5317-7.00002-3

[28] Gelatt KJ Neuwirth L Hawkins DL Woodard JC Hemangioma of the distal phalanx in a colt. Vet Radiol. (1996) 37:275–80. 10.1111/j.1740-8261.1996.tb01230.x

[29] Vos JH Van Der Gaag I Van Dijk JE Wouda W. Lobular capillary haemangiomas in young horses. J Comp Pathol. (1986) 96:637–44. 10.1016/0021-9975(86)90060-5 3819044

